# Dermatofibrosarcoma Protuberans of the Scalp Mimicking Trichilemmal Cyst: A Case Report

**DOI:** 10.7759/cureus.39315

**Published:** 2023-05-21

**Authors:** Thamer H Alsharif, Amin Gronfula, Abdulkarim T Alanazi, Ahmed Deif, Ahmed A Fouda, Hesham Aboueleneein

**Affiliations:** 1 Neurosurgery, The Royal College of Surgeons in Ireland, Dublin, IRL; 2 Orthopaedics, The Royal College of Surgeons in Ireland, Dublin, IRL; 3 School of Medicine, The Royal College of Surgeons in Ireland, Dublin, IRL; 4 Department of Surgery, Section of Neurosurgery, King Fahad Armed Forces Hospital, Jeddah, SAU

**Keywords:** graft, local excision, skin neoplasm, scalp, dermatofibrosarcoma protruberans

## Abstract

We present a case of a 47-year-old female with a swelling on her scalp that was at first thought to be trichilemmal cysts. After two years, she returned to her general practitioner with a larger scalp mass. Following a biopsy, histological analysis revealed dermatofibrosarcoma protuberans (DFSP). She then had the tumor completely removed, resulting in clean margins.

## Introduction

Dermatofibrosarcoma protuberans (DFSP) is a rare cutaneous soft tissue sarcoma that is locally aggressive. It has an incidence rate of 0.8-4.5 cases per million people per year, and it accounts for approximately 6% of soft tissue sarcomas [1]. The majority of DFSPs are low-grade, with the remainder containing a high-grade sarcomatous component [2]. It usually affects the middle layer of skin, the dermis, as well as the subcutaneous fat, and rarely muscle and fascia [3,4]. Although the possibility of metastasis in DFSP is slow, it is characterized by a strong tendency toward local recurrence after surgical resection [5]. Approximately 50% of DFSP cases are in the trunk and proximal extremities, with the head and neck accounting for less than 15% of cases but with a higher recurrence rate than the trunk and proximal extremities [2,6]. The definitive diagnosis requires tissue histology after excisional biopsy, and the literature has established that surgical resection is the mainstay of DFSP treatment in general [4,5]. The preferred initial treatment for localized DFSP is resection with pathologically negative margins. The most effective surgical procedure is determined by the size and location of the tumor [4,5].

We present the case of a 47-year-old female who had a painless soft mass on her scalp misdiagnosed as trichilemmal cysts instead of DFSP.

## Case presentation

**Figure 1:**
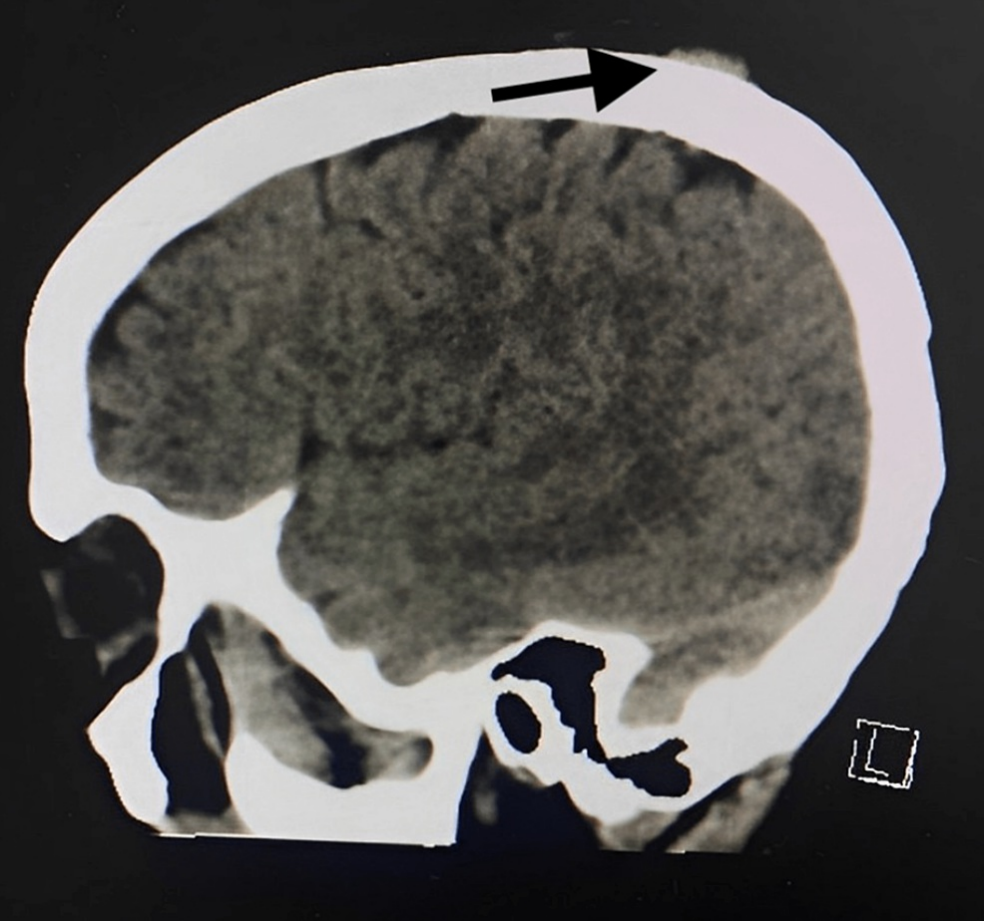
CT scan of the brain without contrast (sagittal view) showing right scalp swelling without bone destruction or intra-cranial extension

**Figure 2:**
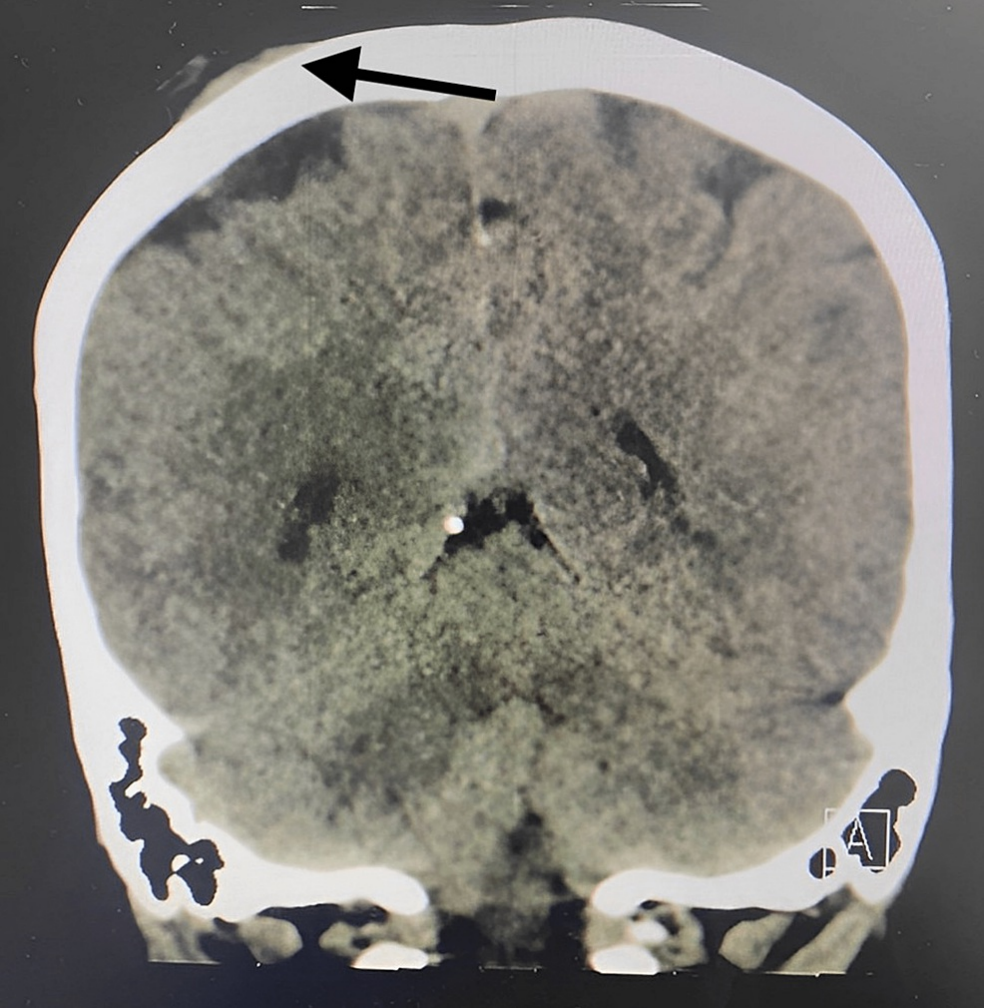
CT scan of the brain without contrast (coronal view) showing right scalp swelling without bone destruction or intra-cranial extension

In January 2021, a 47-year-old female presented to the family medicine clinic with a headache and swelling on the right side of her scalp. A nonenhanced computed tomography (CT) scan of the brain revealed the interval development of a small subcutaneous soft tissue density measuring 1.5 x 0.5 cm in the right parietal region (Figures 1, 2), which was diagnosed as trichilemmal cysts. The excision of the cyst was not arranged.

**Figure 3:**
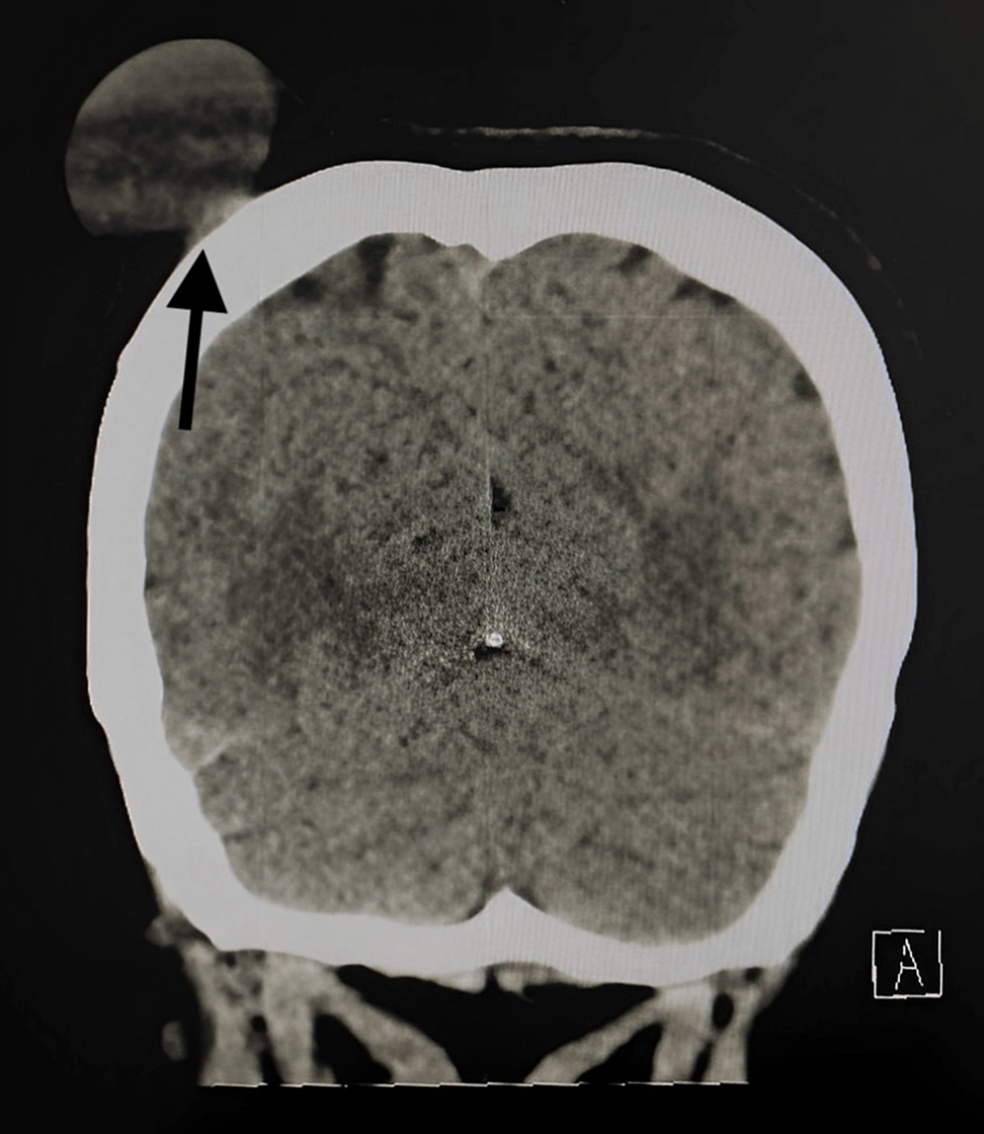
CT scan of the brain without contrast (coronal view) showing significant right scalp swelling with no evidence of bony destruction

**Figure 4:**
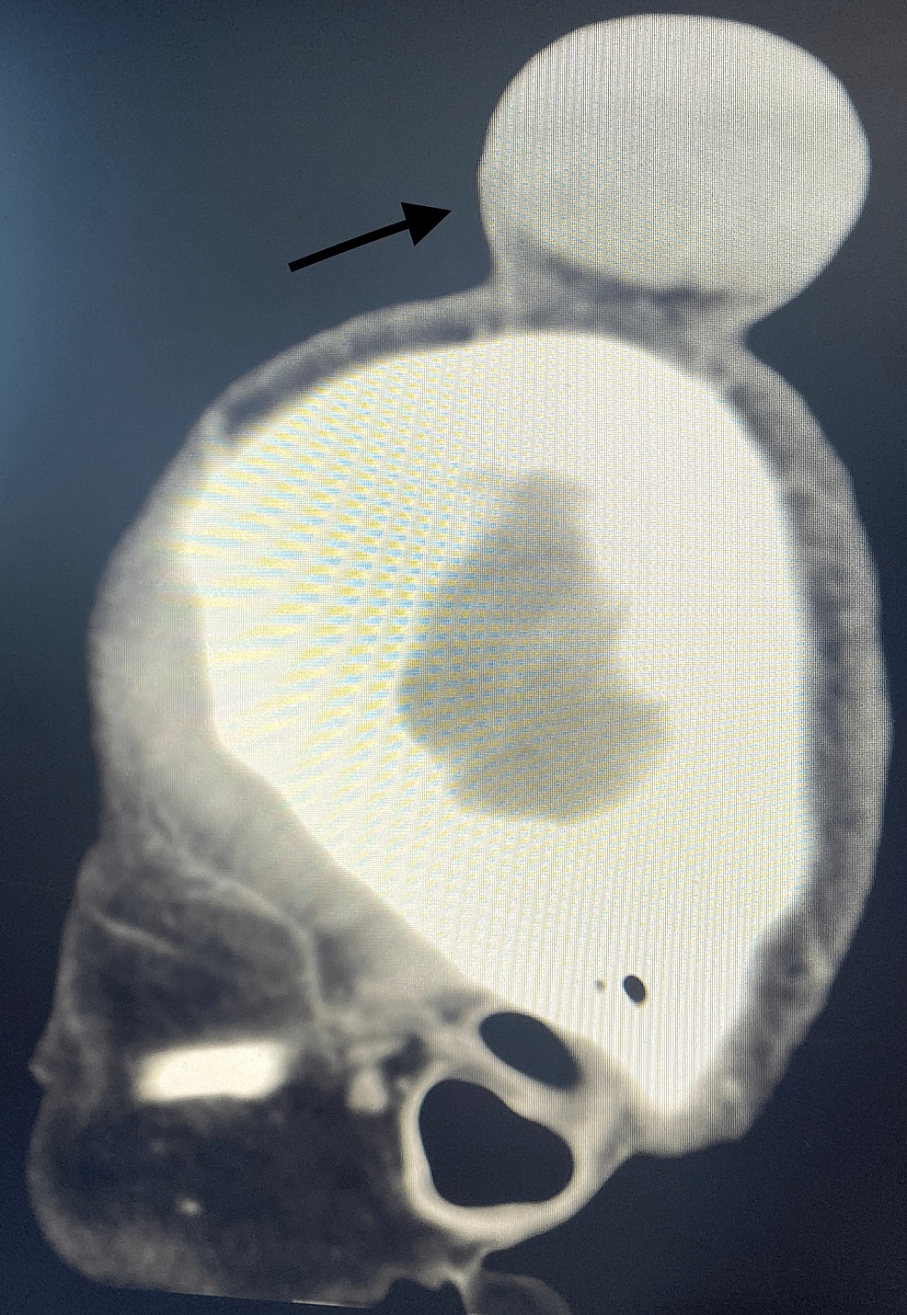
CT scan of the brain without contrast (sagittal view) showing significant sized right scalp swelling with no evidence of bony destruction

She presented to the neurosurgery clinic two years later with a two-year history of chronic headaches and a large, rapidly increasing swelling of the right parietal region over the previous six months. On physical examination, she was conscious, alert, and oriented (Glasgow Coma Scale (GCS) 15/15) with no neurological deficit. The swelling was firm in consistency, painless, mobile, overlaying the skin, and without slippery edges. It measured 3.5 x 4.3 cm with no sign of inflammation or a history of recent trauma. A CT scan of the head showed a significant increase in the size of the swelling in the right parietal region, measuring 6 x 3.5 x 4.5 cm in anteroposterior view with no evidence of a solid component; the underlying parietal bone appeared grossly unremarkable with no evidence of bony destruction (Figures 3, 4).

An excisional biopsy was then performed, and a tissue sample was sent for histopathological analysis. DFSP was diagnosed after the interdepartmental consultation in surgical pathology, and complete excision with margins was advised. An urgent referral to oncology for a metastatic workup was made. CT of the chest, abdomen, and pelvis with IV contrast and a bone scan all revealed no definite signs of solid organ, pulmonary, or bone metastasis. A month later, the patient was admitted and underwent wide excision of the lesion, and the defect on the scalp and neck was simply covered with a graft from a donor area. Her postoperative period was uneventful, and she was discharged from our hospital.

## Discussion

DFSP is a rare soft tissue neoplasm first identified by Sherwell and Taylor in 1890. Darier and Ferrand described it as a progressive recurrent dermatofibroma in 1924. One year later, Hoffmann named it DFSP; DFSP had been reported under a variety of names, including keloid-like sarcomatous tumors, hypertrophic morphea, progressive and recurring dermatofibroma, fibrosarcomatous minors with attenuated dermal surfaces, and skin fibrosarcoma [6,7]. It is thought to have originated from a fibroblastic, muscular, or neurologic undifferentiated mesenchymal cell or dermal stem cell [8].

DFSP has no known cause yet; however, skin injury, tattoos, and pre-existing scars have been suggested as potential risk factors [4]. It can occur at any age and affect any part of the body; it is rarely congenital, although it is most often reported in adults in their mid-thirties [2]. The incidence among women was 1.14 times higher than among men [9]. When DFSP first appears on the skin, symptoms include a bump or growth resembling a pimple that is not tender or painful [10]. It is distinguished by nodular or plaque-like growth that is extremely infiltrative and locally destructive. The lesions are usually reddish, flat, elevated, and firm, with irregular borders or a multinodular appearance, frequently covered clinically by skin-colored, brown-yellow, red-tinged, sclerodermiform, or telangiectatic atrophic skin [10]. The diagnosis of DFSP is suspected clinically and can be confirmed by pathology. Hematoxylin and eosin staining typically reveal diffuse infiltration of the dermis and subcutaneous fat by densely packed, cytologically relatively uniform, spindle-shaped, CD34-positive tumor cells arranged in a distinctive storiform shape [11]. Other histopathology-based differential diagnoses include benign plaque-like CD34-positive dermal fibroma and dermatomyofibroma, pleomorphic sarcoma, leiomyosarcoma, and leiomyosarcoma; however, appropriate and confirmatory immunostainings (CD34, factor XIIIa, stromelysin-3) are highly suggestive of DFSP [7].

Even though metastasis is uncommon, a metastatic workup, including a CT scan of the chest, abdomen, and pelvis, a lymph node USG, and a chest x-ray, should be performed. In some cases, a preoperative MRI may be useful [7]. A treatment plan for DFSP usually includes total excision, during which the surgeon removes the tumor and some healthy tissue from the area. By removing this tissue, cancer that may have spread to a healthy area can be detected earlier. Clear margins have to be achieved before definitive cosmetic reconstriction occurs [7].

## Conclusions

DFSP is a rare, slowly growing tumor that arises from the dermis. It usually occurs in the trunk or proximal extremities. Tumors on the scalp are relatively uncommon in clinical practice. Due to its rarity, misdiagnosis might occur, and a complete excision with a safe margin is usually delayed, and only local excision is performed. Surgeons should be aware of that to improve the health status and outcomes of those patients. In addition, due to the relatively high rate of local recurrence, follow-up should be highly encouraged.
